# IgA Nephropathy: Beyond the Half-Century

**DOI:** 10.3390/medicina60010054

**Published:** 2023-12-27

**Authors:** Yoshio Shimizu, Yasuhiko Tomino, Yusuke Suzuki

**Affiliations:** 1 Division of Nephrology, Department of Internal Medicine, Juntendo University Shizuoka Hospital, 1129 Nagaoka, Izunokuni 410-2295, Shizuoka, Japan; 2 Shizuoka Research Center for Disaster Medicine, Juntendo University, Izunokuni 410-2295, Shizuoka, Japan; 3 Asian Pacific Renal Research Promotion Office, Medical Corporation SHOWAKAI, 3-12-12 Nishishinjuku, Shinjuku-ku, Tokyo 160-0023, Japan; 4 Department of Nephrology, Faculty of Medicine, Juntendo University, 3-1-3 Hongo, Bunkyo-ku, Tokyo 113-8421, Japan; yusuke@juntendo.ac.jp

**Keywords:** IgA nephropathy, RASS-Is, tonsillectomy, glucocorticoid, novel therapeutic targets, RCT

## Abstract

In 1968, Jean Berger first introduced the medical world to IgA nephropathy (IgAN). Fifty-five years later, its pathogenesis is still unclear, but treatments such as renin–angiotensin–aldosterone system inhibitors (RAAS-Is), tonsillectomies, and glucocorticoids are currently used worldwide. There have been great strides in the past 20 years since the discoveries of the specific dysregulation of mucosal immunity, galactose-deficient IgA1 (Gd-IgA1), and Gd-IgA1 immune complexes in patients with IgAN. According to these findings, a multi-hit hypothesis was developed, and this multi-hit hypothesis has provided several putative therapeutic targets. A number of novel agents, including molecularly targeted drugs for targets such as APRIL, plasma cells, complement systems, and endothelin, are undergoing clinical trials. Some candidate drugs have been found to be effective, with minimal side effects. Over half a century after the discovery of IgAN, these therapies will soon be available for clinical use.

## 1. Introduction

IgAN, a prevalent form of primary glomerulonephritis (GN), is distinguished by the accumulation of IgA antibodies within the glomeruli. Within 10-to-20 years of being diagnosed, approximately 20–40% of patients progress to end-stage renal disease (ESRD) [1,2,3]. The discovery of galactose-deficient IgA1 (Gd-IgA1) in patients led to the proposal of a multi-hit hypothesis for the etiology of IgAN [4,5,6]. In this review, we discuss Gd-IgA1, the multi-hit hypothesis, and treatments for IgAN based on those currently used in emerging strategies.

## 2. Materials and Methods

A narrative review was undertaken. We accessed the National Library of Medicine, National Center for Biotechnology Information (PubMed.gov), on 15 June 2023. A literature search was performed using the keywords “IgA nephropathy”, “treatment”, and “options”. The literature search was completed on 13 July 2023. The selected studies came in the form of RCTs, clinical trials, intervention studies, and observational studies. Some good-quality reviews were also selected for new drugs in development for which information is scarce. The selected studies were limited to English-language studies. Among the studies obtained, 8 articles focused on renin–angiotensin–aldosterone system inhibitors (RAAS-Is) (Table 1), 4 articles focused on tonsillectomy (Table 2), 2 articles focused on sodium glucose transporter 2 (SGLT2) inhibitors (Table 3), 4 articles focused on glucocorticoid (Table 4), and 8 articles focused on upcoming therapeutic options (Table 5). Supportive references for review were also selected from PubMed.

## 3. Gd-IgA1 and the Multi-Hit Hypothesis

**Figure 1:**
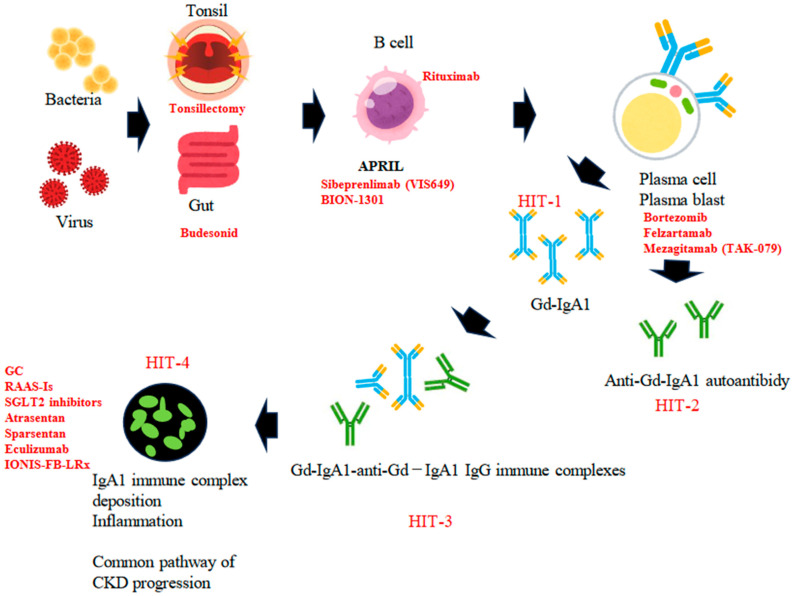
The putative pathogenesis of IgAN, based on the four-hit hypothesis. Hit 1: Initial production of Gd-IgA1 in secondary lymphoid tissues (tonsil or Peyer’s patch in the gut) and transfer of Gd-IgA1-producing cells into bone marrow. Hit 2: Production and circulation of anti-Gd-IgA1 IgG. Hit 3: Formation of Gd-IgA1-anti-Gd-IgA1 IgG immune complexes. Hit 4: Deposition of Gd-IgA1 immune complexes in glomeruli induces focal inflammation. Key molecules or cells and drugs in each step are shown.

The multi-hit hypothesis posits that the production of Gd-IgA1 is the initial hit, which is then followed by the creation of IgG autoantibodies that recognize Gd-IgA1, constituting the second hit. The formation of immune complexes serves as the third hit, and their subsequent deposition in the glomeruli is the fourth hit. This sequence results in hematuria, proteinuria, and a decline in renal function [37,38] (Figure 1).

This comprehension of IgAN’s autoimmune pathogenesis has paved the way for the discovery of various pharmacological therapeutic targets, encompassing the immune response, mucosal immunity, renal inflammation, and complement activation. Therapies based on these mechanisms have been investigated in both preclinical and clinical studies [39].

## 4. Current Therapeutic Options for IgAN

Renal disorders (the pathogenesis of which is described above) can be treated with various therapies, and these therapies have been the subject of clinical studies that have led to the accumulation of evidence of their efficacy. The currently available treatments include renin–angiotensin–aldosterone system inhibitors (RAAS-Is), tonsillectomies, sodium glucose co-transporter 2 (SGLT2) inhibitors, and glucocorticoids.

### 4.1. RAAS-Is

The RAAS is a key player in maintaining blood pressure and volume balance, and its inhibition with ACE inhibitors (ACE-Is) has proven to be an effective treatment strategy in conditions such as hypertension and congestive heart failure. The prevention of angiotensin II formation may also be advantageous in decelerating the progression of renal disease by decreasing glomerular hydrostatic pressure. This presents a hopeful therapeutic avenue for patients with IgAN, potentially enhancing outcomes and decelerating disease progression [40].

Valsartan, an angiotensin receptor blocker (ARB), has been found to significantly reduce proteinuria and slow down renal deterioration in IgAN patients [7], while enalapril, an ACE-I, has demonstrated significant improvements in renal function in proteinuric IgAN [8]. A randomized controlled trial (RCT) that tracked IgAN patients for 5 years to study the long-term renal outcomes of ACE-I/ARB therapy discovered that the treated group had lower serum creatinine, reduced proteinuria, and slower progression to ESRD compared to the control group [9]. One study on the impact of ACE-Is on tubulointerstitial fibrosis (TIF) in IgAN patients observed a slower rate of decline in creatinine clearance in the ACE-I group [10]. A controlled trial examining the response of IgAN patients to ACE-I/ARB therapy based on reduced proteinuria and its effect on the selectivity index suggested that this treatment could be beneficial for IgAN patients with renal impairment and non-selective proteinuria [11].

A combined treatment approach using prednisolone and losartan has been shown to be more effective than using prednisolone alone in decreasing proteinuria and preserving renal function in IgAN patients [12]. The combination of trandolapril and candesartan cilexetil has also been found to be more effective than verapamil in reducing the count of urinary podocytes, which could be an indicator of disease activity in adult IgAN patients [13]. Low-dose losartan significantly diminished proteinuria and urinary N-acetyl-beta-D-glucosaminidase (NAG) excretion without affecting systemic blood pressure in normotensive IgAN patients [14]. These results suggest that these medications could have a positive impact on the management of IgAN.

### 4.2. Tonsillectomies

IgAN patients frequently exhibit macroscopic hematuria following an acute tonsillar infection, leading to the proposition of a tonsillectomy as a potential treatment for IgAN. In Japan, tonsillectomies are employed in conjunction with steroid pulse therapy, and this combination has demonstrated promising outcomes. One RCT involving IgAN patients with proteinuria and low serum creatinine revealed that those who underwent a tonsillectomy and received glucocorticoid pulse therapy had significantly reduced urinary protein excretion compared to those who were administered only a glucocorticoid pulse [15]. Logistic regression analysis identified tonsillectomy and glucocorticoid pulse therapy to be significant independent factors contributing to the elimination of proteinuria. One patient in the tonsillectomy group (*n* = 40) and three patients in the no-tonsillectomy group (*n* = 40) developed diabetes during observation. No other serious complications were observed [15].

A retrospective cohort study conducted in Japan, which included 1065 IgAN patients enrolled between 2002 and 2004, implemented 1: 1 propensity score matching (to mitigate intergroup differences) for patients who underwent a tonsillectomy and those who did not. Among 153 matched pairs, the study revealed a reduced risk of the first instance of a 1.5-fold increase in serum creatinine from baseline or the initiation of dialysis in patients who were treated via a tonsillectomy. These patients also needed fewer additional treatments for one year following renal biopsy without an elevated risk for adverse events, barring temporary complications related to tonsillectomy. These findings imply that tonsillectomy could be a viable option for preventing ESRD in IgAN patients. In the entire study population (*n* = 1065), 59 patients had complications such as pneumonia and diabetes. No deaths occurred in the tonsillectomy group, but four deaths from malignancy, one death from obstructive lung disease, and one death from aortic dissection were observed in the no tonsillectomy group [16].

Tonsillectomies have mainly been performed in Japan, and they have been shown to be effective. In Europe, the efficacy of tonsillectomies has been questioned by many. A Hungarian study of 246 patients showed a significant prolongation of renal survival in the group of patients who underwent a tonsillectomy compared to the group of patients who did not have a tonsillectomy. This study also showed that tonsillectomy was a significant factor for prolonged renal survival [17]. On the other hand, a sub-analysis of the VALIGA study (described below) conducted in Europe to evaluate the efficacy of steroids showed no effect of tonsillectomies on the preservation of renal function [18]. In light of these circumstances, the KDIGO (2021) practice guidelines state that tonsillectomies may be considered in the treatment of Japanese patients [41].

### 4.3. SGLT2 Inhibitors

DAPA-CKD trial: The DAPA-CKD trial demonstrated a reduction in the risk of transitioning to ESRD and mortality in patients with CKD, including those with IgAN. The primary endpoint was a persistent decrease in eGFR of ≥50%, ESRD, or death due to kidney disease-related or cardiovascular causes. Among the 270 IgAN subjects, 137 were administered dapagliflozin, and 133 were given a placebo. The primary outcome was observed in 4% of the dapagliflozin group and 15% of the placebo group. Compared to the placebo group, dapagliflozin reduced UACR by 26%. No significant difference was observed between the two groups in terms of medication discontinuation due to adverse drug reactions. These findings suggest that dapagliflozin can safely slow down the progression of CKD in IgAN patients. A total of six complications in the dapagliflozin group (*n* = 137) forced the discontinuation of the study, while in the sham group (*n* = 133), seven complications forced the discontinuation of the study (no significant difference). Severe complications, including death, occurred in 22 patients (16.1%) in the dapagliflozin group and 34 (25.6%) in the sham group [19].

The EMPA-KIDNEY trial expanded the range of CKD-causing diseases in which SGLT2 inhibitors could be expected to provide benefit. The primary endpoint of the EMPA-KIDNEY trial was a sustained ≥40% reduction in eGFR from basal values occurring after allocation, the introduction of renal replacement therapy (eGFR < 10 mL/min/1.73 m^2^), and death from cardiovascular events. Serious AEs included six cases of ketoacidosis in the empagliflozin group (0.09 patient years) and one in the sham group (0.02 patient years), as well as leg amputations in 28 patients (0.43 patient years) in the empagliflozin group and 19 patients (0.29 patient years) in the sham group. The trial was stopped early due to confirmed efficacy, limiting the analysis to all prespecified subgroups [20]. Therefore, compared to the DAPA-CKD study, the effect of dapagliflozin on IgA nephropathy was not clearly demonstrated. However, the number of patients with CKD due to glomerular disease was larger than in the DAPA-CKD study, 1669/25% (DPA-CKD study: 695/16.9%), and a large number of IgA nephropathy patients would be expected to be among these patients, indirectly suggesting an effect on IgA nephropathy patients [42].

A meta-analysis revealed that SGLT2 inhibitors diminish the risk of renal and cardiovascular events in patients with conditions such as heart failure, CKD, type II diabetes, and atherosclerotic cardiovascular risk. When compared to a placebo, SGLT2 inhibitors decreased the risk of advancing to renal disease by 37% in patients with diabetes (relative risk (RR), 0.63; 95% CI, 0.58–0.69), exhibiting a similar risk reduction effect in non-diabetic patients. Furthermore, SGLT2 inhibitors reduced the risk of acute kidney injury by 23% (0.77, 0.70–0.84) and the risk of death or hospitalization due to heart failure by 23% (0.77, 0.74–0.81) [43].

### 4.4. Glucocorticoids

Early studies have shown that glucocorticoids have long-term renoprotective effects. One study found that patients treated with intravenous methylprednisolone (mPSL) and oral prednisolone had a significantly lower rate of reaching endpoints of a 1.5- or 2-fold increase in baseline serum creatinine compared to controls. No serious AEs, such as hyperkalemia, were recorded [21]. Another study showed that oral prednisolone was renoprotective compared to controls over an 8-year observation period. No serious AEs were observed in either the combination treatment group or the ramipril monotherapy group [22].

(1) VALIGA study: The VALIGA observational cohort study analyzed data from 1147 IgAN patients from 13 European countries. The effects of various treatments, including immunosuppressive agents, on renal outcomes were assessed in a median follow-up period of 4.7 years. The results showed that immunosuppressive agents were associated with a lower risk of renal function decline (> 50% decrease in eGFR or ESRD) than no treatment or antihypertensive drugs, especially in patients with severe histological lesions or high-risk clinical features [23].

(2) STOP-IgAN trial: The STOP-IgAN trial, a randomized controlled trial (RCT), examined the effects of supportive care (SC) alone (including RAAS-Is) versus SC plus immunosuppression (IS) (corticosteroids or cyclophosphamide/azathioprine) in 162 IgAN patients with persistent proteinuria (>0.75 g/day) despite 6 months of optimized RAAS blockade. The trial did not find a significant difference between the two groups in terms of the primary endpoint of full clinical remission (proteinuria < 0.2 g/day and stable renal function) at 3 years. However, the combination of SC and IS was linked to a higher rate of partial remission (proteinuria < 0.5 g/day and stable renal function) and a slower rate of renal function decline (>15 mL/min/1.73 m^2^ decrease in eGFR) compared to SC alone. The immunosuppressed group had significantly more cases of infection and weight gain than the supportive care group, and one patient died of sepsis [24]

(3) TESTING and TESTING 2.0 trial: The TESTING trial was carried out on Chinese patients with IgAN who had urinary protein excretion exceeding 1 g/day and eGFR ranging from 20 to 120 mL/min/1.73 m^2^. The group receiving SC plus IS was administered oral mPSL for a duration of 2 months, and this treatment was gradually tapered off over a period of 6–10 months. The incidence of the primary composite endpoint was significantly lower in the SC plus-IS group compared to the SC-alone group (*p* = 0.02). The SC plus-IS group also exhibited lower urinary protein excretion at the endpoint (*p* < 0.01) and more favorable secondary endpoints (*p* < 0.01, *p* = 0.01, *p* = 0.01). However, the occurrence of adverse effects and severe infection was significantly higher in the SC plus-IS group (*p* < 0.01), leading to a shortened observation period of 2.1 years instead of 3 years [25]. Therefore, this trial found that mPSL treatment had limited benefits. However, mPSL was associated with a greater reduction in proteinuria than SC alone.

The TESTING trial demonstrated the renoprotective effect of the glucocorticoids in IgAN, but this conclusion was considered uncertain due to the shortened observation period and adverse effects of mPSL. Consequently, the TESTING 2.0 trial, with its international, multicenter, and double-blind design, was conducted on patients receiving full-dose (*n* = 136) and reduced-dose (*n* = 126) mPSL. The control patients were given a placebo. A total of 503 patients were randomized and treated with mPSL or the placebo for 2 months and weaned for 6–9 months. Over an average of 4.2 years, the composite primary endpoint occurred in 28.8% of the mPSL patients and 43.1% of the placebo patients [HR 0.53 (95% CI 0.39–0.72); *p* < 0.01]. The effect of full-dose mPSL was an HR of 0.58 (95% CI, 0.41–0.81), and that of reduced-dose mPSL was an HR of 0.27 (95% CI, 0.11–0.65). Nine out of eleven secondary endpoints favored mPSL treatment, including kidney failure (HR, 0.59 (95% CI, 0.11–0.65)). Adverse events were higher in cases treated with mPSL, especially in the full-dose group [26]. Therefore, oral mPSL reduced the risk of kidney function decline, kidney failure, or death due to kidney disease in high-risk IgAN cases but increased the incidence of serious adverse events, mainly with high-dose mPSL.

(4) NEFIGAN and NefIgArd trial: Budesonide (Nefecon^®^) is an oral glucocorticoid engineered to selectively release in the distal ileum, an area densely populated with Peyer’s patches. The NEFIGAN phase IIb double-blind placebo-controlled trial demonstrated that budesonide use resulted in a 24.4% reduction from baseline in the urinary protein-to-creatinine ratio (UPCR) compared to a placebo. Two out of thirteen serious adverse events were potentially linked to budesonide [27]. In November 2021, the US FDA granted Tarpeyo (budesonide), a targeted-release formulation capsule, accelerated approval to decrease proteinuria in adults with primary IgAN who are at risk of rapid disease progression. As part of the conditions for the accelerated approval, a study on Tarpeyo is currently underway to confirm that the drug slows down kidney function decline in patients with IgAN [44].

The NefIgArd trial was an international, phase 3, randomized, double-blind, placebo-controlled multicenter study that aimed to examine the efficacy and safety of 16 mg of Tarpeyo (budesonide) once daily versus a placebo in adult patients with primary IgAN as an adjunct to optimized RAAS-Is therapy. In this study, the initial endpoint of 16 mg of Nefecon for 9 months achieved a significant reduction in UPCR compared to the placebo and also showed a sustained reduction in UPCR after 12 months of treatment. This study also demonstrated a second endpoint of 9 months of Nefecon treatment, a significant retention of eGFR compared to the placebo. The trial also showed that Nefecon was generally well tolerated. No serious AEs due to Nefecon were observed; all cases were minor, and all subjects recovered [28].

After being taken orally and absorbed, budesonide is metabolized at a rate of 90% during its first pass through the liver. This process results in the formation of 6β-hydroxybudesonide and 16α-hydroxyprednisolone. However, these metabolites possess less than 1% of the corticosteroid activity compared to the original compound, budesonide [45]. Given the characteristic pharmacokinetics of budesonide, the following questions require consideration in light of the interim results: (1) Is the intestinal tract the site of pathophysiologically relevant Gd-IgA1 production? (2) Does budesonide change systemic immunity by modifying the intestinal microbiota? (3) Do small amounts of absorbed budesonide have a systemic effect?

## 5. Upcoming Therapeutic Options for IgAN

### 5.1. Therapeutic Target: APRIL

(1) Sibeprenlimab (VIS649): Sibeprenlimab, previously known as VIS649, is a monoclonal antibody that neutralizes A Proliferation-Inducing Ligand (APRIL) and is currently being developed by Visterra Inc. for the treatment of IgAN. A worldwide phase 2 RCT of Sibeprenlimab is in progress to evaluate the efficacy, safety, and tolerability of multiple doses of this drug in patients with IgAN, as well as to assess the dose response to varying doses of Sibeprenlimab by measuring proteinuria. The results from this trial will lay the groundwork for the future clinical development of Sibeprenlimab. Neither the VIS649 group nor the sham group had any serious AEs that resulted in the discontinuation of the study. AEs requiring treatment occurred in 11 patients (39.3%) in the VIS649 group (*n* = 28) and 4 patients (50%) in the sham group (*n* = 8) [29].

(2) BION-1301: BION-1301 is a monoclonal antibody against APRIL. In basic studies, BION-1301 was found to bind to a specific epitope on APRIL and to be able to completely inhibit APRIL-mediated receptor activity. BION-1301 is being investigated as a potential therapeutic option for IgAN. It works by eliminating Gd-IgA1, a pathogenic variant of IgA, and decreasing proteinuria. Currently, BION-1301 is undergoing a phase 1/2 clinical trial with IgAN patients. Early results derived from studying the initial group of IgAN patients indicate that BION-1301 has demonstrated good tolerability and has not resulted in any serious adverse events or the discontinuation of treatment due to side effects [30].

### 5.2. Therapeutic Target: Plasma Cells

(1) Bortezomib: Bortezomib, a proteasome inhibitor, is being examined for its potential to decrease proteinuria in IgAN. A pilot trial (NCT01103778) assessed the impact of four doses of bortezomib on eight consecutive IgAN patients with significant proteinuria. At the one-year follow-up mark, complete remission with urinary protein excretion of less than 300 mg/day, which was the primary outcome, was achieved by three subjects (38%). One of the participants in the study had a T score of 0 according to the Oxford classification prior to entering the study. Among the remaining five subjects, one withdrew within one month of entering the study, and four (50%) either showed no response or experienced disease progression. These findings imply that proteasome inhibition with bortezomib can reduce proteinuria in some IgAN patients, particularly those with a T score of 0 according to the Oxford classification [31].

(2) Felzartamab: Felzartamab is an anti-human CD38 monoclonal antibody found in the MorphoSys HuCAL antibody library. Felzartamab is expected to improve renal function by targeting CD38 and thereby eliminating CD38-positive plasma cells. A phase 2 IGNAZ clinical trial of felzartamab for IgAN has commenced, with the first patient having received his/her dose. This trial, which is a multicenter, randomized, double-blind, parallel-group, placebo-controlled study, aims to enroll approximately 48 patients. Its objective is to evaluate the efficacy, safety, pharmacokinetics, and pharmacodynamics of felzartamab in patients with IgAN [32].

(3) Mezagitamab (TAK-079): Mezagitamab (TAK-079) is a fully human monoclonal antibody (IgG1) that targets the CD38 transmembrane glycoprotein found on the surface of tumor cells, leading to the depletion of these cells through antibody- and complement-dependent cytotoxicity. Initially developed for the treatment of multiple myeloma, this drug is currently being tested in a clinical trial involving adults with primary IgAN who are on stable background therapy. The primary objective of this trial is to evaluate the short-term and long-term side effects of Mezagitamab [33].

### 5.3. Therapeutic Target: Complement Systems

The alternative pathway is initiated by the cleavage of Factor B by Factor D, leading to the activation of additional C3. The lectin pathway is activated by the complexation of pattern-recognition molecules with MASP-3. Both pathways are suggested to be activated in disease progression, as indicated by changes in plasma MASP-3 and the glomerular deposition of Factor H and Factor H-related proteins. These pathways are being explored as potential targets for new treatments for IgAN [46].

(1) Eculizumab: Eculizumab (Soliris^®^) is a humanized recombinant monoclonal IgG antibody against human complement C5. Eculizumab inhibits responses at the end of the complement activation pathway. It was initially developed for the treatment of complement-mediated paroxysmal nocturnal hemoglobinuria (PNH) and atypical hemolytic uremic syndrome (aHUS) [47]. Eculizumab, which selectively binds to C5, prevents the formation of C5a and MAC by inhibiting C5 cleavage. On the other hand, it does not prevent reactions mediated by upstream C3a and C3b. A case report in the literature detailed the case of a patient who experienced the rapid progression of IgAN, leading to renal failure despite undergoing immunosuppressive treatment. In an effort to salvage renal function, the patient was administered eculizumab (anti-C5) for a duration of 3 months. Eculizumab, a C5-neutralizing antibody, inhibits the formation of MAC and thus increases the risk of bacterial infections. The primary bacterial infection is meningitis caused by *Neisseria* meningitidis, but reports of meningitis caused by *Pseudomonas aeruginosa* also exist [48,49]. Eculizumab is already in clinical use in many countries, but its high cost is a problem. Until this problem is resolved, it is unlikely to be administered as a novel treatment for patients with IgAN [50].

(2) IONIS-FB-LRx: IONIS-FB-LRx is an antisense oligonucleotide that reduces the expression of factor B. It is currently being examined in two phase 2 clinical studies involving 25 patients with IgAN. One study involves the drug being administered to 10 patients for 29 weeks, and the other study’s subjects are undergoing dose modification. The results for the first dose cohort of this clinical study were reported at the 2022 American Society of Nephrology (KIDNEY WEEK). The primary endpoint of the study, urinary protein excretion in 24-h urine storage, was reduced by 44%. All subjects completed the protocol, although one subject had a reversible ALT elevation [34].

### 5.4. Therapeutic Target: Endothelin

Endothelin (ET) is an important endogenous vasoconstrictor, a polypeptide composed of 21 amino acids. ET1 is the most potent of the three isoforms and is the only one expressed in the kidney. Prolonged vasoconstriction by ET1 induces glomerular hyperfiltration, which is seen in early diabetic nephropathy and obesity-related nephropathy, and subsequently damages glomerular epithelial cells, causing proteinuria and a reduction in glomerular filtration rate [51].

(1) Atrasentan: Atrasentan, a potent and selective antagonist of the endothelin A receptor, could potentially be beneficial in IgAN and other proteinuric glomerular diseases by decreasing proteinuria. The AFFINITY study is a phase 2 international study aiming to determine the efficacy and safety of administering atrasentan to IgA patients with proteinuria, Alport syndrome, focal glomerulosclerosis, and diabetic kidney disease (DKD). The interim results through week 24 of treatment for the 20 patients in the IgAN cohort of the AFFINITY study showed that 79% achieved a >40% reduction in proteinuria [52]. The ALIGN study, a phase 3, double-blind, placebo-controlled trial, is currently investigating the efficacy and safety of atrasentan in patients with IgAN who are at risk of progressive renal function loss. A total of 16 subjects had mild AEs, but none was severe enough to cause the stopping of the study [35].

(2) Sparsentan: The PROTECT phase 3 clinical trial, currently in progress, is investigating the effectiveness and safety of a unique non-immunosuppressive single-molecule drug called sparsentan. This drug is a dual antagonist for both endothelin and angiotensin receptors and is being tested on adults with IgAN. The trial is being carried out across 18 countries at 134 clinical practice sites. An interim analysis, predetermined for the primary proteinuria efficacy endpoint, revealed that sparsentan significantly reduced proteinuria compared to a control group that received irbesartan. The safety profile of sparsentan was found to be comparable to that of irbesartan. No cases of serious edema or heart failure were observed, and no cases were discontinued due to AEs [36].

## 6. Conclusions

Table 6 summarizes the treatment options for IgA nephropathy. The treatments can be divided into two main categories: immune and non-immune.

In the past, glucocorticoids, which extensively suppress the immune system, have been used in the treatment of the immune system and have been shown to decrease proteinuria and suppress renal function decline, but serious side effects, such as infection, have been a problem. However, with the development of new therapeutic agents and their clinical application, therapies that target molecules on B cells, complement, etc., are attracting attention. Although these agents have not yet been fully tested in clinical trials, interim reports indicate that they are effective in reducing proteinuria and have milder side effects than glucocorticoids.

On the other hand, RAAS-Is, involved in non-immune therapies, have been shown to reduce proteinuria and be renoprotective. In addition, SGLT2 inhibitors for type 2 diabetes have been shown to be effective in non-diabetic CKD and are expected to provide new treatment options for IgAN. In addition, anti-endothelin agents such as atrasentan and sparsentan are also expected to be renoprotective because of their ability to decrease proteinuria, and they are also expected to have minimal side effects. Since these therapies have different mechanisms of action, synergistic effects from their combination are also expected.

Although the pathogenesis of IgAN is not yet fully understood, new treatments are being developed; more than 50 years after IgAN was first discovered, more effective treatments with fewer side effects are being developed. As research continues to advance, we can expect to find even more effective treatments.

## Figures and Tables

**Table 1:** RAAS-Is.

Author (Year), Location	Design	Population (Sample Size, Age, Health)	Methods	Health Outcomes	Main Findings
Li et al. (2006), Hong Kong [7]	Multicenter RCT	*n* = 109, age > 18 years Group 1: UP > 1 g/day and S-Cr < 2.8 mg/dL. Group 2: S-Cr 1.4–2.8 mg/dL	Treatment group (*n* = 54): valsartan 80–160 mg/day. Control group (*n* = 55): placebo for 104 weeks.	GFR and UP	Treatment group: decreased 33% of UP. Mean decrease rate of GFR was less than that of control group. Control group: no significant change in UP
Praga et al. (2003), Spain [8]	Single-center RCT	*n* = 44. Age: treatment group, 27.8 ± 12 years; control group, 29.9 ± 12.3 years. UP < or = 0.5 g/day and S-Cr < 1.5 mg/dL.	Treatment group (*n* = 23): dose arrangement of for BP under 140/90 mmHg with enalapril. Control group (*n* = 21): dose arrangement for BP under 140/90 mmHg with other than ACE-I or ARB. Mean follow-up period: 78 ± 28 months.	Primary endpoint: 50% increase in basal s-Cr. Secondary endpoint: s-Cr > 1.5 mg/dL and increase in UP.	Treatment group: decreased 33% of UP. Mean decrease rate of GFR was less than that of control group. Control group: No significant change in UP. In total, 13% of Pt in enalapril group reached primary endpoint and 52% in control group. Proteinuria significantly decreased in enalapril group.
Woo et al. (2007), Singapore [9]	Single-center RCT	*n* = 75. Age: 62 ± 5 years. UP > 1 g/day and/or S-Cr > 1.6 mg/dL.	Treatment group (*n* = 37): enalapril 5–10 mg/day or losartan 50–100 mg/day. Control group: no treatment. Follow-up: 5 years.	UP, S-Cr, progression to ESKD	The study suggests that the common mechanism of therapy is likely through lower levels of ACE, glomerular pressure, and proteinuria, resulting in reduced renal damage and slowed progression to ESKD.
Kanno et al. (2005), Japan [10]	Prospective 3-year follow-up study	*n* = 49. Age: ACE-I group, 35 ± 2 years; CCB group, 35 ± 1 years.	ACE-I group (*n* = 26): dose arrangement BP < 130/85 mmHg with trandolapril or temocapril. CCB group (*n* = 23): dose arrangement with BP < 130/85 mmHg with amlodipine 2.5–5 mg/day	CCr	In the ACEI group, the rate of decline in CCr was lower.
Woo et al. (2000), Singapore [11]	Case control trial	*n* = 41. Age: treatment group, 39 ± 10 years; control group, 37 ± 6 years. UP > 1 g/day and/or S-Cr > 1.4 mg/dL.	Treatment group (*n* = 21): dose arrangement BP < 150/90 mmHg with enalapril 5–10 mg/day or losartan 50–100 mg/day. Control group (*n* = 20): dose arrangement BP < 150/90 mmHg with other than ACE-I, ARB or CCB.	UP, Selectivity Index (SI), Ccr	Among the 21 patients in the treatment group, 10 responded to ACEI/ATRA therapy with a decrease in proteinuria by 30% (responders). Among the responders, SI improved. Eight out of twenty-one patients in the treatment group who had documented renal impairment had improved renal function compared with two in the control group.
Horita et al. (2007), Japan [12]	Single-center RCT	*n* = 38. Age: 33 ± 11 years. BP < 140/90 mmHg. UP, 1.0–1.6 g/day; and CCr > 50 mL/min/1.73 m^2^.	Treatment group (*n* = 20): prednisolone 30 mg/day (2 mo), 20 mg/day (2 mo), 15 mg/day (6 mo), 10 mg/day (12 mo) and 5 mg/day (1 mo) and losartan 50 mg. Control group (*n* = 18): prednisolone as treatment group. Follow-up period: 2 years.	UP, Ccr	Both groups showed a significant decrease in proteinuria compared to baseline. However, the combination of PSL plus LST was more effective than PSL alone. The group treated with PSL plus LST maintained a similar creatinine clearance level, while the group treated with PSL alone showed a decrease.
Nakamura et al. (2000), Japan [13]	Non-blinded controlled trial Trial	*n* = 32. Age: mean 32.6 years (18–54), normotensive (BP < 140/90 mmHg), UP < 3 g/day and Ccr > 80 mL/min.	Verapamil group (*n* = 8): 120 mg/day, trandolapril group (*n* = 8): 2 mg/day, candesartan group (*n* = 8): 8 mg/day. Control group (*n* = 8): placebo. Follow-up period: 3 mo.	UP, urinary podocytes	The antiproteinuric response in the trandolapril group was similar to that in the candesartan cilexetil group, and both were greater than that of verapamil. The reduction in the number of urinary podocytes from baseline was significantly greater in patients treated with trandolapril or candesartan cilexetil than in patients treated with verapamil.
Shimizu et al. (2008), Japan [14]	Single-center RCT	*n* = 36. Normotensive (BP < 140/90 mmHg), mild proteinuria.	Treatment group: losartan (*n* = 18) 12.5 mg/day Control group: anti-platelet agent	UP, S-Cr, urinary NAG	Low-dose losartan significantly reduced proteinuria from 0.8 +/− 0.5 g/d at baseline to 0.4 +/− 0.4 g/d at 12 months (*p* = 0.006). Proteinuria was significantly lower at 12 months in the losartan group than in the control group (*p* = 0.04). Urinary N-acetyl-beta-D-glucosaminidase (NAG) levels at 12 months were significantly lower in the losartan group than in the control group (*p* = 0.009).

Abbreviations: RCT, randomized controlled trial; S-Cr, serum creatinine; UP, urinary protein; CCr, creatinine clearance; ACE-I, angiotensin-converting enzyme inhibitor; ACE, angiotensin-converting enzyme; ATRA, angiotensin II receptor antagonist; PSL, prednisolone; ESKD, end-stage kidney disease.

**Table 2:** Tonsillectomy.

Author (Year), Location	Design	Population (Sample Size, Age, Health)	Methods	Health Outcomes	Main Findings
Kawamura et al. (2014), Japan [15]	Multicenter RCT	*n* = 72. Age: 10–69 years. UP: 1.0–3.5 g/day and S-Cr < 1.5 mg/dL.	Group A (*n* = 33): tonsillectomy and steroid pulse therapy. Group B (*n* = 39): tonsillectomy. Observation period: 12 mo.	UP, hematuria, clinical remission	UP was significantly reduced in the patients with tonsillectomy and steroid pulse therapy. No significant difference in the attenuation of hematuria and the rate of clinical remission. AE: *n* = 54 (total), death (*n* = 6) in Group B due to malignancy (*n* = 2), COLD (*n* = 1), aortic dissection (*n* = 1)
Hirano et al. (2016), Japan [16]	Observational study	*n* = 1065. Age (median): 35 (25–52) years. Propensity score-matched analysis.	Propensity-matched patiemts. Tonsillectomy group (*n* = 153). Control group (*n* = 153): RAAS-Is and/or steroids. Observation period: 3.6 years.	1.5-fold increase in serum creatinine level from baseline or dialysis initiation.	In a matching analysis, tonsillectomy was associated with primary outcome reduction (hazard ratio, 0.34; 95% CI, 0.13–0.77; *p* = 0.009).
Kovács et al. (2014), Hungary [17]	Retrospective cohort study	*n* = 264. Age: group without tonsillectomy, 38 ± 113.9 years; group with tonsillectomy, 30.2 ± 10.1 years. CKD stage G1-G3.	Group without tonsillectomy (*n* = 166): RAAS-Is and/or statin and/or steroid. Mean observation period: 133 ± 102 mo. Group with tonsillectomy (*n* = 98): tonsillectomy, RAAS-Is, statin, steroid. Mean observation period: 171 ± 114 mo.	Renal survival time	The mean renal survival time was significantly longer in patients who underwent tonsillectomy. Tonsillectomy, baseline eGFR, and 24-h proteinuria were identified as independent risk factors for renal end points.
Feehally et al. (2016), Europe [18]	Retrospective cohort study (sub-analysis of VALIGA study)	*n* = 1147. Age: tonsillectomy group, 31.1 ± 17.9 years; control group (without tonsillectomy), 36.0 ± 16.5 years. Propensity-matched analysis.	Tonsillectomy group (*n* = 41) and control group (*n* = 41): RAAS-Is and/or steroids. Observation period > 4.7 years.	Impact of tonsillectomy on the progression to ESKD and/or a 50% loss of estimated glomerular filtration rate (eGFR).	No significant difference in outcomes was seen between the two groups. This was also the case when pairing patients who underwent tonsillectomy after the diagnosis of IgAN with those who did not have the procedure.

Abbreviations: RCT, randomized controlled trial; CKD, chronic kidney disease; UP, urinary protein; RAAS-Is, renin–angiotensin–aldosterone inhibitors; ESKD, end-stage kidney disease; eGFR, estimated glomerular filtration rate; COLD, chronic obstructive lung disease; AE, adverse effect; CI, confidence interval.

**Table 3:** SGLT2 inhibitors.

Author (Year), Location	Design	Population (Sample Size, Age, Health)	Methods	Health Outcomes	Main Findings
Wheeler et al. (2021), worldwide [19]	Multicenter RCT (sub-analysis of DAPA-CKD trial)	*n* = 270. Age: 51.2 years (mean). UACR, 900 mg/gCr; and mean eGFR, 43.8 mL/min/1.73 m^2^	Dapagliflozin group (*n* = 137): dapagliflozin 10 mg/day Placebo group (*n* = 133) Follow-up period: 2.4 years (median)	Primary outcome: sustained decline in eGFR of 50% or more, end-stage kidney disease, or death from a kidney disease-related or cardiovascular cause. UCR, decline rate of eGFR,	The primary outcome occurred in 4% of participants on dapagliflozin and 15% on placebo. Dapagliflozin also reduced the UACR by 26% relative to placebo. The mean rates of eGFR decline with dapagliflozin and placebo were −3.5 and −4.7 mL/min/1.73 m^2^/year, respectively. AE leading to discontinuation of study drug: dapagliflozin group, 6/137; placebo group, 7/133. Severe AEs, including death: dapagliflozin group, 22(16.1%); placebo group, 34(25.6%).
Nuffield et al. (2022), worldwide [20]	Multicenter RCT (EMPA-KIDNEY trial)	*n* = 6609, CKD patients. Age (mean): 63.9 years (empagliflozin group) and 63.8 (placebo group), eGFR 25 ≤ 45 mL/min/1.73 m^2^, UACR > 200 mg/gCr 853 (26%) and 816 (25%) patients with glomerular disease in empagliflozin group and placebo group, respectively.	Empagliflozin group (*n* = 1097): empagliflozin 10 mg/day. Placebo group (*n* = 1095). Follow-up period: 2.0 years (median).	Primary outcome: end-stage kidney disease, a sustained eGFR < 10 mL/minute/1.73 m^2^, a sustained decline in eGFR of ≥40%, or a renal death) or death from cardiovascular causes.	The 13.1% of patients in the empagliflozin group experienced progression of kidney disease or death from cardiovascular causes, compared to 16.9% in the placebo group. This result was consistent among patients with or without diabetes and across different levels of kidney function. AE: Ketoacidosis: 6/1 (0.09/0.02 patient-years, lower-limb amputation: 28/19 (0.43/0.29 patient-years) in empagliflozin/placebo group, respectively.

Abbreviations: DAPA, dapagliflozin; RCT, randomized controlled trial; Cr, creatinine; UACR, urinary albumin-to-creatine ratio, eGFR, estimated glomerular filtration rate; AEs, adverse events.

**Table 4:** Glucocorticoids.

Author (Year), Location	Design	Population (Sample Size, Age, Health)	Methods	Health Outcomes	Main Findings
Lv et al. (2009), China [21]	Single-center RCT	*n* = 63, age > 18–65 years. Group 1: UP 1–5 g/day and eGFR > 30 mL/min/1.73 m^2^	Combination group (*n* = 33): cilazapril 2.5→5 mg/day and 6-to-8-month course of prednisone was started with oral prednisone, 0.8-to-1.0 mg/kg/d, for 8 weeks; the dose was tapered by 5 to 10 mg every 2 weeks. Cilazapril group (*n* = 30): 2.5→5 mg/day for 6–8 mo. Follow-up period: 48 mo.	Primary endpoint: defined as a 50% increase in baseline serum creatinine level UP.	The 7 patients in the cilazapril group (24.1%) reached the primary end point compared with 1 patient (3%) in the combination group. UP was significantly decreased in patients in the combination group compared with the cilazapril group (time-average proteinuria, 1.04 ± 0.54 versus 1.57 ± 0.86 g/d of protein; *p* = 0.01). Severe AEs, including hyperkalemia, were not observed.
Manno et al. (2009), Italy [22]	Single-center RCT	*n* = 97. Age: ramipril-alone group, 34.9 ± 11.2 years; prednisone plus-ramipril group: 31.8 ± 11.3 years. Histological grade: moderate. UP > or = 1.0 g/day, and eGFR > or = 5 mL/min/1.73 m^2^	Ramipril-alone group (*n* = 23): dose arrangement for BP under 120/80 mmHg with ramipril (starting with 2.5 mg/day). Control group (*n* = 21): dose arrangement for BP under 120/80 mmHg with ramipril (starting at 2.5 mg/day). 6-month course of prednisone began with oral prednisone 1.0 mg/kg/day for 2 months and then the dose was tapered by 0.2 mg/kg/day every mo. The maximal prednisone dose was fixed at 75 mg/day. Follow-up period: 96 mo.	Primary endpoint: Combination of doubling of baseline serum creatinine or ESKD, defined as a need for dialysis or renal transplantation. Secondary endpoint: Decline by means of eGFR slope over time and UP.	The13/49 (26.5%) patients in the ramipril alone group reached the primary outcome compared with 2/48 (4.2%) in the prednisone plus-ramipril group. The mean rate of eGFR decline was higher in the ramipril alone group than in the combination therapy group (−6.17 ± 13.3 vs. −0.56 ± 7.62 mL/min/1.73 m^2^). Prednisone plus-ramipril treatment reduced 24-h proteinuria more than ramipril alone during the first 2 years. Severe AEs were not observed in both groups.
Tesar et al. (2015), Europe [23]	Retrospective cohort study (sub-analysis of VALIGA study, propensity matched)	*n* = 1147. Age: 36 ± 16 years (mean). UP, 1.3 g/day (0.6–2.6). eGFR,, 73 ± 30 mL/min/1.73 m^2^. Caucasian: 97%. MEST score, mesangial hypercellularity (M1) was present in 28%, endocapillary hypercellularity (E1) was present in 11%, segmental glomerulosclerosis (S1) was present in 70%, and 21% of the patients had >25% tubular atrophy and interstitial fibrosis (T1–2).	Supportive care group (*n* = 80): corticosteroid and RAAS-Is. Corticosteroid plus supportive-care group: (*n* = 82) RAAS-Is. Observation period: 4.7 years.	50% decrease in eGFR or ESKD	Corticosteroid reduced proteinuria and the rate of renal function decline and increased renal survival. These benefits extended to those with an eGFR </= 50, and the benefits increased proportionally with the level of proteinuria. Corticosteroid reduced the risk of progression regardless of initial eGFR and in direct proportion to the extent of proteinuria in this cohort.
Rauen et al. (2015), Europe [24]	Multicenter RCT (STOP-IgAN study)	*n* = 162, Age: 43.7 ± 12.8 years (mean), UP > 0.75 g/day	Supportive care group (*n* = 26): dose arrangement BP < 125/75 mmHg with RAAS-Is. Immunosuppression group (*n* = 23): Dose arrangement with BP < 125/75/85 mmHg with RAAS-Is and glucocorticoid monotherapy for 6 mo (methylprednisolone, administered intravenously at a dose of 1 g per day for 3 days at the start of months 1, 3, and 5; and oral prednisolone at a dose of 0.5 mg per kilogram per 48 h on the other days). Follow-up period: 3 years.	Primary endpoints: Full clinical remission at the end of the trial (protein-to-creatinine ratio < 0.2 [with both protein and creatinine measured in grams] and a decrease in the estimated glomerular filtration rate (eGFR) of <5 mL per minute per 1.73 m^2^	The 4 patients (5%) in the supportive-care group, as compared with 14 (17%) in the immunosuppression group, had a full clinical remission (*p* = 0.01). A total of 22 patients (28%) in the supportive-care group and 21 (26%) in the immunosuppression group had a decrease in the eGFR of at least 15 mL per minute per 1.73 m^2^ (*p* = 0.75). More patients in the immunosuppression group than in the supportive-care group had severe infections, impaired glucose tolerance, and weight gain of more than 5 kg in the first year of treatment. One patient in the immunosuppression group died of sepsis.
Lv et al. (2017), worldwide [25]	Multicenter RCT (Testing trial)	*n* = 272. Age: 38.6 ± 11.1 years. (mean). UP > 1 g/day. eGFR: 20-to-120 mL/min/1.73 m^2^ after at least 3 mo of blood pressure control with RAAS-Is.	Treatment group (*n* = 136): oral methylprednisolone (0.6–0.8 mg/kg/d; maximum, 48 mg/day for 4–6 mo. Control group (*n* = 126): placebo for 4–6 mo. The mean required follow-up period: 5 years.	Primary renal outcome: ESKD, death due to kidney failure, or a 40% decrease in eGFR.	The study stopped early (after 28 of the 335 planned events) due to a significantly increased risk of serious adverse events with oral methylprednisolone vs. placebo (14.7% vs. 3.2% primarily excess infections). The primary renal outcome occurred in 8 participants (5.9%) in the methylprednisolone group vs. 20 (15.9%) in the placebo group (hazard ratio, 0.37 (95% CI, 0.17–0.85); risk difference, 10.0% (95% CI, 2.5–17.9%); *p* = 0.02).
Lv et al. (2022), worldwide [26]	Multicenter RCT (Testing 2.0 trial)	*n* = 503. Age: 38 years (mean), UP > 1 g/day. eGFR: 20-to-120 mL/min/1.73 m^2^ after at least 3 mo of blood pressure control with RAAS-Is	Participants were randomized in a 1:1 ratio to receive oral methylprednisolone (initially 0.6–0.8 mg/kg/d, maximum 48 mg/d, weaning by 8 mg/d/mo; *n* = 136) or placebo (*n* = 126). After 262 participants were randomized, an excess of serious infections was identified, leading to dose reduction (0.4 mg/kg/d, maximum 32 mg/d, weaning by 4 mg/d/mo) and addition of antibiotic prophylaxis for pneumocystis pneumonia for subsequent participants (121 in the oral methylprednisolone group and 120 in the placebo group). The mean required follow-up: 5 years.	The primary composite outcome: ESKD, death due to kidney failure, or a 40% decrease in eGFR. Predefined safety outcomes were serious infection, new diabetes, gastrointestinal hemorrhage, fracture/osteonecrosis, and cardiovascular events.	In this randomized clinical trial that included 503 participants, a 6-to-9-month course of oral methylprednisolone, compared with the placebo, significantly reduced the risk of the composite outcome of kidney function decline, kidney failure, or death due to kidney disease (hazard ratio, 0.53); however, the risk of serious adverse events was increased. Severe AEs were observed in 28 (10.9%) patients in methylprednisolone group and 7 (2.8%) in placebo group.
Fellström et al. (2017), Europe [27]	Multicenter RCT Phase 2b (NEFIGAN trial)	*n* = 149. Age: mean 39 ± 12.3. years. Persistent proteinuria despite optimized renin–angiotensin system (RAS) blockade.	TRF-budesonide 8 mg/day (*n* = 51), TRF-budesonide 16 mg/day (*n* = 48), Placebo (*n* = 50). Follow-up period: 9 mo.	Primary outcome: mean change from baseline in UPCR. Safety was assessed in all patients who received the intervention.	Mean UPCR decreased by 27.3% in 48 patients who received 16 mg/day (0.71; 0.53–0.94; *p* = 0.0092) and 21.5% in the 51 patients who received 8 mg/day (0.76; 0.58–1.01; *p* = 0.0290); 50 patients who received placebo had an increase in mean UPCR of 2.7%. The incidence of adverse events was similar in all groups (43 [88%] of 49 in the TRF-budesonide 16 mg/day group, 48 [94%] of 51 in the TRF-budesonide 8 mg/day, and 42 [84%] of 50 controls). AEs: 13 cases of AEs were recorded. Deep vein thrombosis (16 mg/day) and unexplained deterioration in renal function in follow-up were considered to be TRF-budesonide related.
Barratt et al. (2023), worldwide [28]	Multicenter RCT Phase 3 (NefIgArd trial)	*n* = 199. Age 44 years (Nefecon group), 43 (placebo group) (mean), UPCR >/= 0.8 g/gCr, or UP > 1 g/day, eGFR >/= 35, </= 90 mL/min/1.73 m^2^	Treatment group: (*n* = 97) Nefecon 16 mg/day. Control group: (*n* = 100) placebo.	Primary outcome: UPCR after 9 mo. Secondary outcome: eGFR after 9 and 12 mo.	At nine months, UPCR was 27% lower in the Nefecon group compared with placebo, along with a benefit in eGFR preservation corresponding to a 3.87 mL/min/1.73 m^2^ difference versus placebo (both significant). Nefecon was well tolerated, and treatment-emergent adverse events were mostly mild-to-moderate in severity and reversible.

Abbreviations: RCT, randomized controlled trial; UP, urinary protein; mo, months; ESKD, end-stage kidney disease; eGFR, estimated glomerular filtration rate; UPCR, urinary protein-to-creatinine ratio; CI, confidence interval; AEs, adverse events.

**Table 5:** Upcoming therapeutic options for IgAN.

Author (Year), Location	Drug (Target)	Design	Population (Sample Size, Age, Health)	Methods	Health Outcomes	Main Findings
Mathur et al. (2022), USA [29]	VIS649 (APRIL)	Phase 1 randomized, placebo-controlled, single ascending-dose, first-in-human study	*n* = 45, age: 18–55 years, BMI: 18–32, IgG > 750 mg/dL, IgA > 80 mg/dL, IgM > 55 mg/dL, healthy volunteer.	Placebo (*n* = 8): Single injection VIS649 0.5 mg/Kg (*n* = 7), 2 mg/Kg (*n* = 7), 6 mg/Lg (*n* = 7), 12 mg/Kg (*n* = 7): single injection	Standard safety assessments, including AEs, clinical laboratory tests, vital signs, electrocardiograms, and physical examinations, were performed at regular intervals. The PK profile of VIS649, the effect of VIS649 on various PD parameters.	There were no serious adverse events (AEs). Half-life increased with dose, and drug exposure increased in a greater than dose-proportional manner. Serum APRIL, IgA, galactose-deficient (Gd) IgA1, IgG, and IgM were reversibly suppressed in a dose-dependent manner, with a dose–response in time to recovery. Treatment-emergent AEs (TEAEs) were experienced by 4 of 8 (50.0%) participants who received placebo and 11 of 28 (39.3%) who received VIS649 (all doses).
Barratt et al. (2021), UK [30]	BION-1301 (APRIL)	Phase1/2, Part 1: double-blind, randomized, placebo-controlled, single ascending dose (SAD) in healthy volunteers (HVs). Part 2: double-blind, randomized, placebo-controlled multiple ascending dose (MAD) in HVs.	*n* = ?, Age > 18 (18–55) years. eGFR >/= 30 mL/min/1.73 m^2^, UP > 0.5/day or UPCR >/= 0.5 g/gCr, On stable and optimized RAAS-Is treatment	Cohrt 1: BION-1301: 400 mg, iv/every 2 weeks→600 mg, sc/every 2 weeks Cohort2: BION-1301: 600 mg, sc/every 2 weeks	Incidence of Treatment Emergent Adverse Events (TEAEs) and Severity of TEAEs up to 76 weeks. APRIL, UP, UPCR, Immunoglobulin, Gd-IgA1.	No SAEs or terminations due to AEs. Durable reductions in serum levels of free APRIL and immunoglobulins were observed. In Cohort 1, clinically meaningful reductions in proteinuria were seen as early as 12 weeks (30.4% geometric mean UPCR reduction, *n* = 7) and were sustained through 24 weeks (48.8% geometric mean UPCR reduction, *n* = 8) and 52 weeks (66.9% geometric mean UPCR reduction, *n* = 8). Reductions in proteinuria were consistent in Cohort 2 (53.8% geometric mean UPCR reduction, *n* = 9) at 24 weeks. Significant and durable reductions in serum Gd-IgA1 concentrations were observed.
Hartono et al. (2018), USA [31]	Bortezomib (Plasma cell, CD38)	Pilot trial	*n* = 8, Age: 35 ± 12 (22–53) years, UP > 1 g/day and/or CCr > 30 mL/min, stable-dose RAAS-Is.	Bortezomib 1.3 mg/m^2^ (BSA), 4 doses/every 2 weeks Follow-up period: 1 yr	Primary endpoint: clinical remission, UP, S-Cr, UPCR	At 1-year follow-up, 3 subjects (38%) had achieved the primary endpoint. Four patients (50%) did not have any response or had progression of disease. All of the participants tolerated 4 doses of bortezomib without any serious AEs.
Maixnerova et al. (2023), worldwide [32]	Felzartamab (plasma cell, CD38)	Phase 2a, Multicenter RCT	*n* = 48, Age: 18–80 years, UP > 1 g/day, Treatment with RAAS-Is	#1 Placebo, #2 Experimental arm 1: felzartamab, arm 2: felzartamab, arm 3: felzartamab (detail not shown). Follow-up period: 9 mo.	Safety: determined by the frequency, incidence and severity of TEAEs, UP, Complete response, Pharmacokinetic: serum concentrations of Felzartamab over time	Not shown.
Takeda Pharmaceuticals (2023), Japan [33]	Mezagitamab (plasma cell, CD38)	Phase 1b, multicenter, open-label study	*n* = 41, Age: UP > 1 g/day or UPCR > 1 g/gCr, eGFR >/= 45 mL/min/1.73 m^2^	Mezagitamab, subcutaneous injection, once weekly for 8 weeks then once every 2 weeks for 16 weeks in the main study. Follow-up period: 48 weeks	Main Study: Percentage of Participants With one or More Treatment-emergent Adverse Events (TEAEs), Grade 3 or Higher TEAEs, Serious Adverse Events (SAEs), and Adverse Events (AEs) Leading to Mezagitamab Discontinuation.	Not shown.
IONIS Pharma (2022), USA, Asia, Oceania [34]	IONIS-FB-LRx (Complement system, Factor B)	Phase 2, single-arm, open-label clinical study	*n* = 25, Age: 25–62 years. Proteinuria > 1.5 g/day hematuria despite maximum tolerated RAAS blockade. eGFR > 40 mL/min/1.73 m^2^	Participants will receive IONIS-FB-LRx, by subcutaneous injection (SC) at Week 1 and every 4 weeks through Week 25. Optional 48-week Extension, with drug dosing continuing every 4 weeks.	Percent reduction in 24-h urine protein excretion (time frame: baseline to week 29, UP, UACR, Factor B, AH50)	IONIS-FB-LRx met its primary endpoint of change in 24-h urinary protein, demonstrating a 44% mean reduction in proteinuria from baseline to week 29. Kidney function, as measured by estimated glomerular filtration rate (eGFR), was maintained in all patients in the study. IONIS-FB-LRx achieved robust and sustained reductions in plasma complement Factor B (CFB), alternative pathway activity (AH50), and urinary complement fragment Ba (Factor Ba). AE: a reversible elevation of ALT (*n* = 1). All patients completed the study.
Heerspink et al. (2021), worldwide [35]	Atrasentan (endothelin, ET_A_ receptor)	Phase 3 multicenter RCT	*n* = 340. Age: UP > 1 g/day despite taking RAAS-Is, eGFR >/= 30 mL/min/1.73 m^2^, SGLT2 inhibitors: available.	Atrasentan group (*n* = ?): 0.75 mg/day for 132 weeks. Placebo group (*n* = ?): placebo for 132-weeks follow-up period: 3 mo.	UP, UPCR (at week 24), eGFR (at 2.6 years), second composite endpoint: 40% reduction in eGFR, dialysis, transplantation, and all-cause mortality.	Atrasentan demonstrated mean proteinuria reductions of 38.1% proteinuria at six weeks of treatment, 48.3% at 12 weeks of treatment, and 54.7% at 24 weeks of treatment. Atrasentan was generally well tolerated. Treatment-emergent AEs observed in 16 patients were mild or moderate in severity.
Heespink HJL et al. (2023), worldwide [36]	Sparsentan (endothelin, angiotensin receptor)	Multicenter RCT	*n* = 404. Age: sparsentan group, 46.6; irbesartan group, 45.4 years (mean). UP > 1 g/day despite maximum RAAS blockade.	Sparsentan group: (*n* = 202) 400 mg/day for 36 weeks irbesartan group: (*n* = 202) irbesartan 300 mg/day for 36 weeks.	UP, UPCR (at week 36), TEAEs.	Mean percent change from baseline in UPCR was statistically significantly greater in the sparsentan group (–49.8%) than the irbesartan group (–15.1%), resulting in a between-group relative reduction of 41% (least squares mean ratio = 0.59; 95% CI, 0.51–0.69; *p* < 0.0001). TEAEs with sparsentan were similar to irbesartan. There were no cases of severe oedema, heart failure, hepatotoxicity, or oedema-related discontinuations.

Abbreviations: RCT, randomized controlled trial; RAAS, renin–angiotensin–aldosterone; eGFR, estimated glomerular filtration rate; UP, urinary protein; RAAS-Is, RAAS Inhibitors; SGLT2, sodium glucose transporter 2; UACR, urinary albumin-to-creatinine ratio; UPCR, urinary protein-to-creatinine ratio; ALT, alanine amino transferase; AEs, adverse events.

**Table 6:** Summary of the treatment options.

Treatment Option	Therapeutic Target	RCT	UP Reduction	Reno-Protection	AEs	Remarks
RAAS-Is	Intraglomerular pressure	Performed	Shown	Shown	Mild	
	Common pathway of CKD					
	Progression					
Tonsillectomy	Tonsillar lymphoid tissues	Performed	Shown (Japan);	Shown (Japan)	Mild	Available in Japan
			not shown (Europe)	Not shown (Europe)		
Dapagliflozin	Tubular SGLT2	Performed	shown	Shown	Mild	
	Common pathway of CKD					
	Progression					
Empagliflozin	Tubular SGLT2	Performed	Not performed	Shown	Mild	
	Common pathway of CKD					Data from CKD patients
	Progression					IgAN (25%)
	Cellular immunity	Performed	Shown	Shown		* Infection, diabetes, death
Glucocorticoid	Humoral immunity				Moderate–severe	
(prednisolone)	Inflammation				With high dose *	
TRF-budesonide	Gut lymphoid tissues	Performed	Shown	Shown	Severe with high dose *	* Infection, deep vein thrombosis
VIS649	APRIL (B cell)	Performed	Shown	Not shown	Mild	
BION-1301	APRIL (B cell)	Performed	Confirmed	Not shown	Mild	
Bortezomib	Plasma cell (CD38)	Not performed	Shown *	Not shown	Mild	* Patients with T score of 0 on the Oxford classification
Felzartamab	Plasma cell (CD38)	Ongoing *	Not shown	Not shown	Not shown	* Phase 2 study
Mezagitamab	Plasma cell (CD38)	Not performed *	Not shown	not shown	Not shown	* Phase 1 study
Eculizumab	Complement C5	Not performed	Not performed	Not performed	Not performed	Too expensive
IONIS-FB-LRx	Complement Factor B	Ongoing *	Shown	Not performed	Mild	* Phase 2 study interlim results
Atrasentan	Endothelin (ETA receptor)	Performed	Shown	Not performed	Mild–moderate	
Sparsentan	Endothelin (ETA receptor)	Performed *	Shown	Not performed	Mild	* Phase 3 study
	Angiotensin receptor					

Abbreviations: RAAS-Is, renin–angiotensin–aldosterone system inhibitors; SGLT2, sodium–glucose cotransporter 2; RCT, randomized controlled trial; APRIL, A Proliferation-Inducing Ligand; CKD, chronic kidney disease.

## Data Availability

Data sharing is not applicable to this article as no new data were created or analyzed in this study.
